# Intense exercise increases dopamine transporter and neuromelanin concentrations in the substantia nigra in Parkinson’s disease

**DOI:** 10.1038/s41531-024-00641-1

**Published:** 2024-02-09

**Authors:** Bart de Laat, Jocelyn Hoye, Gelsina Stanley, Michelle Hespeler, Jennifer Ligi, Varsha Mohan, Dustin W. Wooten, Xiaomeng Zhang, Thanh D. Nguyen, Jose Key, Giulia Colonna, Yiyun Huang, Nabeel Nabulsi, Amar Patel, David Matuskey, Evan D. Morris, Sule Tinaz

**Affiliations:** 1 Department of Radiology and Biomedical Imaging, Yale University, New Haven, CT USA; 2 Department of Psychiatry, Yale University, New Haven, CT USA; 3 Beat Parkinson’s Today, East Hartford, CT USA; 4 Neuroscience, AbbVie, North Chicago, IL USA; 5 Department of Radiology, Weil Cornell Medicine, New York, NY USA; 6 Department of Biomedical Engineering, Yale University, New Haven, CT USA; 7 Department of Neurology, Yale University, New Haven, CT USA

**Keywords:** Parkinson's disease, Translational research

## Abstract

Parkinson’s disease (PD) is characterized by a progressive loss of dopaminergic neurons. Exercise has been reported to slow the clinical progression of PD. We evaluated the dopaminergic system of patients with mild and early PD before and after a six-month program of intense exercise. Using ^18^F-FE-PE2I PET imaging, we measured dopamine transporter (DAT) availability in the striatum and substantia nigra. Using NM-MRI, we evaluated the neuromelanin content in the substantia nigra. Exercise reversed the expected decrease in DAT availability into a significant increase in both the substantia nigra and putamen. Exercise also reversed the expected decrease in neuromelanin concentration in the substantia nigra into a significant increase. These findings suggest improved functionality in the remaining dopaminergic neurons after exercise. Further research is needed to validate our findings and to pinpoint the source of any true neuromodulatory and neuroprotective effects of exercise in PD in large clinical trials.

## Introduction

Parkinson’s disease (PD) is a neurodegenerative disorder characterized by the loss of dopaminergic neurons in the substantia nigra (SN)^1^. A growing literature has demonstrated the benefits of exercise programs for controlling motor symptoms of PD^2–5^. The reported benefits vary according to the type, intensity, and duration of the exercise. Moderate-to-high-intensity exercise multiple times per week for prolonged periods (e.g., 6 months) has been shown to ameliorate the motor severity of PD in clinical trials^6–8^.

Rodent models of PD have shown that exercise-induced improvements in motor performance were accompanied by neuroprotective effects on the dopaminergic neurons in the SN^9–16^. These neuroprotective effects of exercise are thought to be mediated through neurotrophic, anti-inflammatory, and angiogenic factors. It has been suggested that the interplay between these factors facilitates rescuing of the dopaminergic neurons and increased signaling capacity of healthy dopaminergic neurons^17,18^. In humans, indirect clinical evidence suggests that exercise may be neuroprotective. Exercise studies in patients with PD support the mediator role of neurotrophic and anti-inflammatory factors in clinical improvement^19–21^. Low pro-inflammatory microglial activation has been proposed as a pathway linking physical activity to brain health based on postmortem examination of older adults without PD but with varying degrees of physical activity^22^. Postmortem nigral volumes and white matter integrity have been found to be positively correlated with physical activity in older adults without PD^23^. A positron emission tomography (PET) study using [^11^C]-raclopride in people with PD demonstrated that a single bout of vigorous cycling by habitual exercisers released significantly more dopamine in the caudate nucleus than the same activity performed by those who were sedentary^24^. These findings suggest that the benefits of exercise may be due to neuromodulatory effects, such as the preservation of the dopaminergic reserves and enhanced dopamine transmission. However, these putative effects have not been directly investigated in humans with PD in vivo.

Multimodal neuroimaging enables the visualization and quantification of multiple aspects of the dopaminergic system and its functioning in vivo. Imaging of the dopaminergic system has been used to track disease progression in PD. A recent study using the radioligand ^18^F-FE-PE2I (selective for the presynaptic dopamine transporter (DAT) protein) demonstrated reduced DAT availability in the SN and striatum in subjects with early PD over the course of 2 years, whereas healthy age-matched controls showed no significant changes^25^. Neuromelanin (NM) is a cytosolic neuronal pigment and an autophagic product synthesized via oxidation and polymerization of catecholamines such as dopamine. It accumulates slowly in dopaminergic neurons with age^26^ and loss of NM is a hallmark of PD pathology. NM-sensitive magnetic resonance imaging (MRI) can assay NM in the dopaminergic neurons of the SN. A negative correlation between motor symptom severity and NM-MRI measurements in the SN has been demonstrated in PD^27,28^. In independent PD cohorts, the annual rates of decline in SN volumes were estimated using NM-MRI, suggesting a role for NM as a biomarker for disease progression in the brains of people with PD^28^. Lastly, using iron-sensitive MRI techniques such as Quantitative Susceptibility Mapping (QSM), increased ferromagnetic depositions in the SN have been demonstrated in people with PD^29,30^.

In this proof-of-concept study, we imaged the effects of 6 months of high-intensity interval training on the dopaminergic system in patients with PD. Our primary outcome was the change in DAT availability in the SN and striatum, as measured with ^18^F-FE-PE2I. Our secondary outcome was the change in NM concentration in the SN. We also used QSM to account for potentially confounding effects of ferromagnetic depositions on NM measurements.

## Results

### Subjects

Thirteen subjects were enrolled in the “Beat Parkinson’s Today” high-intensity interval training program. After the initial exercise trial period, two subjects dropped out, one due to scheduling conflicts and the other due to incomplete healing after a foot surgery. One additional subject was excluded from the NM-MRI analysis due to imaging artifacts and from the PET analysis due to inability to schedule the scan within the time window of the study and the inability of the subject to continue with exercise beyond the 6-month period for reasons unrelated to the study. The demographic and clinical data (*n* = 10), exercise data, and pre- and post-exercise motor function test results are summarized in Tables 1, 2, and 3, respectively.

**Table 1:** Demographic and clinical data.

	Pre-exercise	Post-exercise
Age (years)	64.2 ± 5.2	
Sex	6 males, 4 females	
Disease duration (years)	2.0 ± 0.8	
Symptom onset side	5 right, 5 left	
Pre-exercise moderate-intensity exercise (hr/wk)	8.4 ± 4.8	
Pre-exercise moderate-intensity MET/kg/wk	32.3 ± 20.3	
Weight (kg)	76.6 ± 14.5	74.6 ± 14.1
H & Y	2.0 ± 0.0	2.0 ± 0.0
MDS-UPDRS I	8.6 ± 6.1	6.5 ± 4.2
MDS-UPDRS II	3.5 ± 2.2	3.1 ± 1.6
MDS-UPDRS III	27.6 ± 4.4	27.0 ± 8.4
MDS-UPDRS IV	1.0 ± 1.2	0.6 ± 1.0
MDS-UPDRS total	37.6 ± 13.6	37.2 ± 11.0
MoCA	28.1 ± 1.8	28.2 ± 1.1
STAI-T	34.5 ± 11.4	34.5 ± 12.4
BDI-II	7.8 ± 6.6	5.7 ± 5.0
Apathy	10.9 ± 6.8	8.2 ± 5.4
PDQ-39-SI	11.8 ± 7.0	9.2 ± 4.6
PFS-16	2.1 ± 1.2	2.0 ± 1.1
LEDD (mg)	245.0 ± 157.1	215.0 ± 173.3

*BDI-II* Beck Depression Inventory-II, H & Y Hoehn & Yahr disease stage, *LEDD* Levodopa-equivalent daily dose, *MDS-UPDRS* Movement Disorders Society-Unified Parkinson’s Disease Rating Scale (I/II: Nonmotor/motor aspects of experiences of daily living, III: Motor examination, IV: Motor complications), *MoCA* Montreal Cognitive Assessment test, *PDQ-39-SI* The Parkinson’s Disease Questionnaire-39-Summary Index, *PFS-16* Parkinson’s Fatigue Scale-16, *STAI-T* Spielberger Anxiety Inventory-Trait.

**Table 2:** Exercise data.

# Completed exercise classes	86.5 ± 20.2
*Heart rate (HR) data*	
Resting HR (bpm)	65.1 ± 7.6
Max HR (bpm)	153.3 ± 5.4
% classes at >80% max HR target	59.4 ± 36.6
% classes at >70% max HR target	85.6 ± 23.7
*Weekly surveys*	
Motivation to exercise	3.9 ± 0.1
Satisfaction with class/trainer	3.9 ± 0.1
Satisfaction with own performance	3.5 ± 0.4
Intensity of class	3.6 ± 0.3

Scale (0–4) for surveys: 0: very unmotivated, very unsatisfied, not intense at all and 4: very motivated, very satisfied, very intense.

*Bpm* beat per minute.

**Table 3:** Motor function tests.

	Pre-exercise	Post-exercise
5 times sit and stand (s)	9.4 ± 1.7	8.7 ± 1.6
360° turn (s)	2.5 ± 0.8	2.3 ± 0.4
Timed-up-and-go (s)	7.0 ± 1.6	6.5 ± 0.9
Climb up 1 flight of stairs (s)	4.8 ± 0.9	4.8 ± 0.6
2-min walk (m)	182.0 ± 38.1	189.0 ± 30.8

Performance on standard motor function tests is shown (mean ± standard deviation).

On average, our PD cohort had mild bilateral disease, intact global cognition; and no significant anxiety, depression, apathy, or fatigue compared with the normative population data (see supplementary data). One subject was not taking any PD medication, and only six subjects were on L-dopa. Subjects fulfilled the class attendance requirements and reported high levels of motivation to exercise and high satisfaction with the program. Subjects reported only mild and transient side effects of exercise such as muscle soreness and fatigue. In about two-thirds of all classes, the target HR was achieved (i.e., 80% of the maximum HR), however, 90% of classes were rated as very intense by the subjects. Motor function tests showed similar or slightly improved scores from pre- to post-exercise.

Six subjects continued the exercise program beyond the six months period. Their average annual motor exam score (collected at 12.6 ± 2.3 months after the start of exercise in the “off” medication state) was 23.7 ± 6.6, improved from the baseline (26.7 ± 7.2) and unchanged from the 6-month (23.8 ± 7.9) scores.

### Imaging

After the last exercise class, there was a period of 4.9 ± 3.0 days until the MRI scan and 10.1 ± 3.1 days until the PET scan.

### PET

**Fig. 1:**
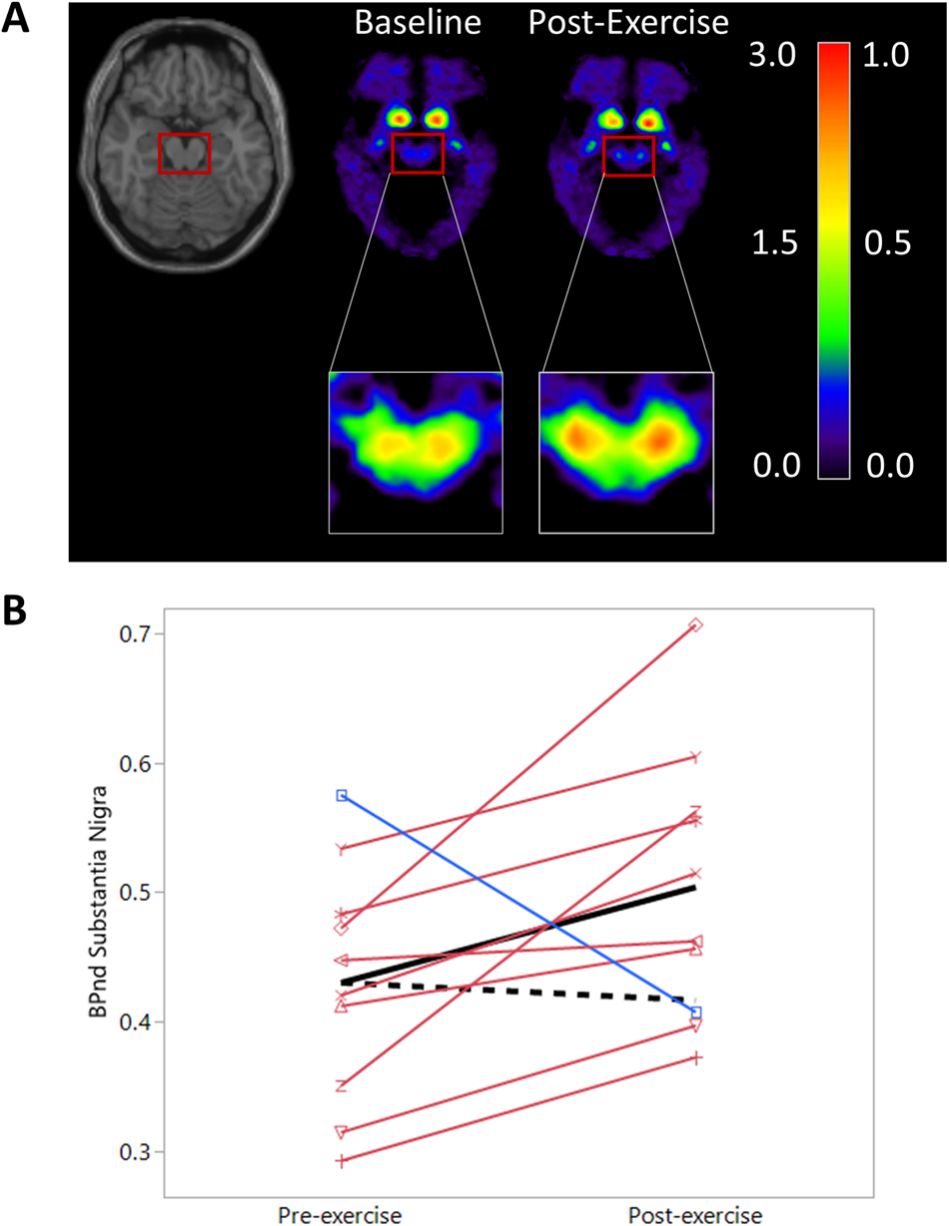
Dopamine Transporter Levels Pre- and Post-Exercise. **A** Average ^18^F-FE-PE2I DAT BP_ND_ images before and after six months of exercise. The red box including the midbrain and SN is enlarged. Note: The left side of the color bar (0.0–3.0) corresponds to the DAT BP_ND_ in the striatum and the right side (0.0–1.0) to the DAT BP_ND_ in the SN shown in the inset images. BP_ND_ is unitless. Orientation is axial. **B** ^18^F-FE-PE2I BP_ND_ in the SN pre- and post-exercise by study participant. Individual lines are red if an increase was observed, blue if a decrease was observed. The solid black line represents the mean of our cohort, the dashed black line represents the expected decrease from the pre-exercise average in the absence of intervention^25^.

The pre- and post-exercise ^18^F-FE-PE2I BP_ND_ values were: Caudate: 0.93 ± 0.11 and 0.95 ± 0.09, putamen: 1.51 ± 0.18 and 1.61 ± 0.27, and SN: 0.43 ± 0.03 and 0.50 ± 0.03 (Fig. 1A).

For the caudate, the observed average of the individual change was a 20.16% increase (90% CI: −21.3–61.6%) was higher than the reported 3.90% *decrease* in BP_ND_ per six months for a similar PD cohort but did not reach significance (*p* = 0.160). For the putamen, the observed 4.32% increase (90% CI: −4.6–13.3%) was significantly higher than the reported 5.35% *decrease* in BP_ND_ per six months (*p* = 0.004). For the SN, the observed 19.95% increase (90% CI: 5.7–34.2%) was significantly higher than the reported 2.25% *decrease* in BP_ND_ per six months (*p* = 0.010) (Fig. 1B).

### NM-MRI and QSM

**Fig. 2:**
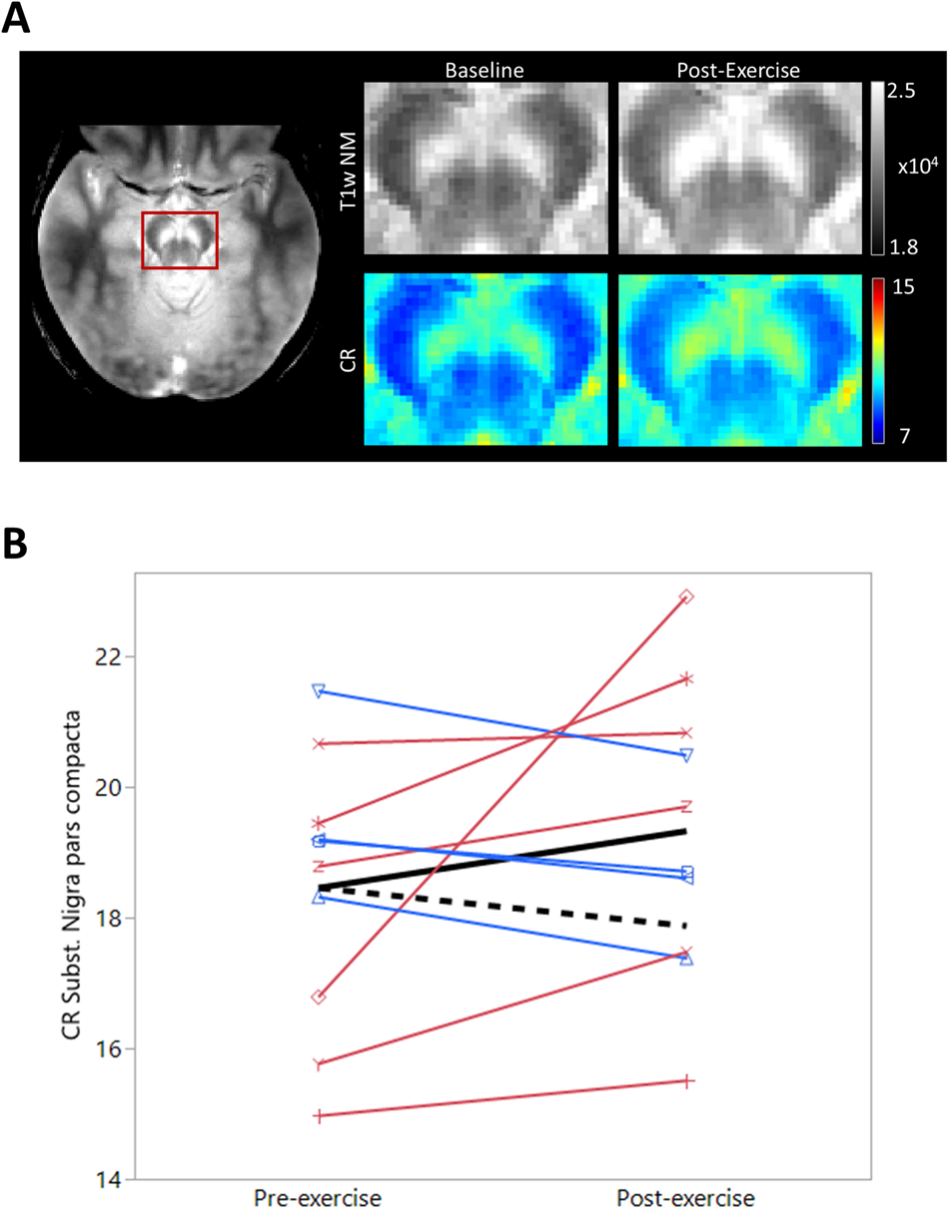
Neuromelanin Levels Pre- and Post-Exercise. **A** Average NM-MRI pre- and post-exercise. Axial image on the left shows the acquired NM image and the red box locates the cut-out sections shown. Top row shows the raw T1-weighted NM image (T1w NM), the bottom row shows the corresponding CR values. **B** CR in the SNc pre- and post-exercise by study participant. Individual lines are red if an increase was observed, blue if a decrease was observed. The solid black line represents the mean of our cohort, the dashed black line represents the baseline average multiplied by the expected decrease without intervention^31^.

The pre- and post-exercise CR values of the SNc were 18.46 ± 0.65 and 19.33 ± 0.71, respectively (Fig. 2A).

The observed 5.3% increase (90% CI: −5.1–34.0) in the SNc is significantly greater (*p* = 0.008) than the 3.15% *decrease* reported for a CR in a similar PD cohort over six months (Fig. 2B). The participant with lower DAT availability in the SN after exercise (blue line, Fig. 1B) is also one of the participants with lower CR post-exercise. No significant difference in the QSM value in SNc was observed (pre-exercise = 131.5, post-exercise = 126.0, paired t-test, *p* = 0.59), suggesting that the observed increases in NM were not confounded by the change in iron deposits.

## Discussion

Six months of high-intensity exercise induced brain changes in patients with early and mild PD. We observed a consistent increase in available DAT sites in the SN. A more variable increase was observed in available DAT sites in the putamen. Using NM-MRI, we observed an increase in the NM signal in the SNc. These apparent increases in DAT and NM in the SN were significantly different from previously observed natural declines in comparable PD populations^25,31^.

The DAT protein is critical in maintaining intracellular dopamine storage^32^. Regulation of the DAT protein homeostasis is particularly complex in PD. In early disease stages, dopamine turnover has been found to be increased. Significantly lower levels of DAT mRNA expression in SN neurons have been found in post-mortem brain tissue in people with PD compared to controls^33,34^. Yet, higher levels of DAT mRNA expression have also been observed in the remaining SN dopaminergic neurons in PD. These changes in DAT homeostasis have been attributed to neuronal dysfunction or compensatory changes in dopaminergic signaling in the remaining neurons. Recently, more than 44% annual reductions in DAT availability in the SN were observed with ^18^F-FE-PE2I by Delva et al. in a comparable cohort with early and mild PD^25^. We found that six months of intense exercise induced a significant increase in DAT availability in the SN in 90% of our participants. Given that there were no signs of motor disease progression (i.e., no worsening of MDS-UPDRS-III scores) and no change in the total levodopa daily dose, the increase in DAT availability likely reflects improved functionality in the remaining dopaminergic neurons post-exercise. These findings are also in line with the neuroprotective effects of aerobic exercise on the SN dopaminergic neurons in rodent models of PD^9–16^.

We found smaller and less consistent increases in DAT availability in the putamen and caudate than in the SN. Post-mortem studies have demonstrated a near total loss of nigrostriatal terminals in the dorsal putamen at 4 years post-diagnosis^35^. Our cohort, restricted to patients with less than 4 years of disease, was followed during a period in which a considerable loss of nigrostriatal terminals would have been expected^25^. The absence of a clear decrease in the striatal DAT availability in our cohort may be seen as evidence of a protective effect of exercise on the nigrostriatal terminals, especially in the putamen.

Neuromelanin accumulates linearly with age in dopaminergic neurons^36^ but decreases up to 60% in the first years of PD^37^. Neuromelanin is thought to play a dual role: (1) when confined to the intracellular space, it can protect the cell against free radicals; (2) when released from dying dopaminergic neurons, it can cause more toxicity by inducing neuroinflammation^28,36^. We observed an increase in NM signal in the SNc which was a significant reversal of the expected decrease in six months reported by Xing et al. in a comparable cohort, but with a longer disease duration (4.9 ± 1.8 years in Xing et al. versus 2.0 ± 0.8 years in our cohort)^31^. Considering that the fastest decline in the nigral NM concentration takes place early in the disease course, we think that the NM signal increase in our cohort is striking. NM-MRI cannot differentiate whether the observed increase in NM concentration in the SNc is intra- or extracellular, but given our clinical and PET findings, the increase in NM signal can reasonably be seen as reflecting greater metabolic/synthetic activity in the remaining dopaminergic neurons.

Our cohort included high-functioning patients with mild PD who were motivated to exercise and to comply with the program. Subjects reached HRs of 70% of their maximum and higher in more than 85% of classes, which is consistent with their rating 90% of their classes as very intense. This suggests that subjects exercised at more than moderate-intensity level (typically, 60–65% of the maximum HR) in most classes. Participants reached the target HR of 80% of their theoretical maximum in two-thirds of classes. The discrepancy between the ratings and HR data may be due to mechanisms such as cardiac sympathetic denervation which is common in PD^38^ and would restrict increases in HR in response to an exercise challenge. Importantly, we did not find any significant motor progression or increase in levodopa equivalent daily dose. Together, these points suggest that our exercise program resulted in clinical benefit despite not always exceeding the prescribed HR threshold. Finally, the 1-year MDS-UPDRS-III scores of a subset of subjects who continued the program were comparable to their 6-month scores suggesting sustained clinical benefit. Similar delays in motor progression have been reported in randomized controlled trials of 6-months of high intensity exercise^6,7^. Our results suggest extension of this benefit over 1 year.

Two main limitations exist in our study. The first is the absence of a control group. A matched PD control group in a mild exercise program would have controlled for the potential confound of positive feedback received by participation in any group activity. It would have allowed for direct measurement of the natural disease progression at hand. Moreover, we only enrolled patients with mild and early PD and tried to minimize attrition by including an exercise trial period before enrollment. While these measures limit the generalizability of our results and may have introduced a selection bias, we think that they were necessary measures to ensure post-exercise signal detection in the brain in this proof-of-concept study. The second limitation is the small sample size. Smaller samples can lead to over- and underestimation of true effects due to the risk of relatively high proportion of extreme cases. But an increase of DAT availability in SN in 9/10 participants is unlikely to have occurred by chance. Related to the small sample size issue, we did not include sex or side of symptom onset as covariates in our analyses. An additional point of concern might be the scheduled break of 1-2 weeks between the end of the exercise sessions and imaging sessions. We included this break as a wash-out period, during which the known acute increase in dopamine concentration after exercise would be expected to disappear and only the longer lasting changes would remain. While we cannot be certain that this break was of appropriate duration, we think that any potential carryover or withdrawal effect from exercise on the imaging data was balanced out because the break duration between the exercise and imaging sessions was approximately the same at baseline and 6 months.

In summary, this proof-of-concept study provides in vivo evidence that sustained periods of intense exercise can induce brain changes in individuals with mild and early PD. Two different biomarkers for the health of the dopamine system were increased in the SN following six months of exercise demonstrating the neuromodulatory effects of exercise on the dopaminergic system. Moreover, the increases were both significantly different from, and reversals of, the expected natural *decline*. These same markers did not decline in the striatum as would also have been expected during the natural course of disease progression. Our results not only support the inclusion of high-intensity exercise early in the treatment plans of PD patients, but also suggest a role for exercise as an effective non-invasive neuromodulatory therapy. Future randomized controlled trials will be needed to optimize exercise regimens. Our observations could also have far-reaching implications for neuroprotective effects of exercise in PD, but further work is needed to validate them and elucidate the underlying mechanisms.

## Methods

### Subjects

This study was designed as a within-subject proof-of-concept. We recruited subjects with mild PD defined according to the Movement Disorders Society (MDS) diagnostic criteria^39^ through the Yale Movement Disorders Clinic and via ‘Beat Parkinson’s Today’ gyms that cater to PD exercisers. Subjects were excluded if they had: Hoehn & Yahr disease stage >2^40^, who were >4 years after diagnosis^35^, had a neurological or psychiatric disorder (other than PD and comorbid depression or anxiety), a medical condition that might affect the central nervous system, history of alcohol or illicit drug abuse, head injury resulting in loss of consciousness, dementia (Montreal Cognitive Assessment (MoCA) score <21)^41^, contraindications for MRI or PET, or high baseline exercise levels (i.e., baseline exercise equals or exceeds the Beat Parkinson’s Today intensity and frequency).

Eligible subjects participated in 5-6 exercise classes for a 2-week trial period. Subjects who could perform the exercises and were committed to continue were enrolled in the 6-month program by giving written informed consent in accordance with the procedures approved by the Yale Human Investigation Committee.

### Clinical evaluations

At the start of the exercise period, clinical evaluations were performed including the Community Healthy Activities Model Program for Seniors (CHAMPS) questionnaire^42^ to determine baseline moderate-intensity physical activity levels, neurological and movement exams using the MDS-Unified PD Rating Scale (MDS-UPDRS)^43^, motor function tests, cognitive evaluation using the MoCA test^41^, and self-evaluation surveys for anxiety^44^, depression^45^, apathy^46^, fatigue^47^, and quality of life^48^. All assessments except CHAMPS were repeated at a post-exercise clinical visit. The motor exams were performed in the medication “off” state (i.e., 12-h washout after the last dose) and videotaped for scoring by a masked movement disorders neurologist (A.P.) (except for rigidity, which was scored by a movement disorders neurologist (S.T.) during the exam). We also performed a motor exam in a subset of subjects after one year. The motor function tests of gross movement speed included the two-minute endurance walking, timed up-and-go, five times sit-to-stand, 360° turning, and climbing one flight of stairs.

During the 6-month exercise period, weekly surveys were filled out by subjects reporting their experience with the program and by trainers reporting their observations about subjects during each class. The trainers and research team addressed issues regarding the subjects and logistics (exercise performance, protocol adherence, injuries, proper use of chest straps, etc.) at monthly meetings.

### ‘Beat Parkinson’s Today’ exercise program

The Beat Parkinson’s Today exercise program was developed for people with PD mirroring the ParkFit exercise program^49^. Beat Parkinson’s Today offers high-intensity interval training and boxing, both of which have been shown to benefit people with PD^50,51^. The high-intensity interval training circuits are designed to improve aerobic capacity, muscle strength and endurance, gait and balance, physical function, and flexibility. Every workout is adjusted to an individual’s needs and ability (e.g., using chairs if kneeling down is difficult). The exercises are performed in small groups in a supportive environment fostering a community bond and compliance. A typical workout includes warm-up (5 min), exercise (30 min) with two consecutive circuits (composed of strength, cardio, and power exercises) each performed twice with 30 s rest periods between rounds, boxing (15 min), and cool-down (10 min). Trainers provided individual feedback to participants and logged class attendance to verify that each subject completed the required number of classes (72 in total). Due to the Covid-19 pandemic, subjects participated in live online Beat Parkinson’s Today classes in the period between January 2021 to August 2022. The online classes were taught by Beat Parkinson’s Today trainers in small groups (5–8 attendees). Trainers monitored the participants in real-time for safety and compliance and provided immediate feedback. From here on, for simplicity, we refer to the Beat Parkinson’s Today exercise regimen as “exercise”.

### Heart-rate chest-strap

Participants wore chest straps with a Polar H7 heart rate (HR) monitor (Polar Electro Oy, Kempele, Finland) starting five minutes before, and continuing throughout every exercise session. The target HR for each participant was defined as 80% of his/her maximum HR. Maximum HR was determined according to the formula: Maximum HR = 220-age. Beat-to-beat interval data were collected with the EliteHRV app and filtered using a Butterworth filter to minimize high frequency artifacts. Average HRs during the 20 min containing the highest mean HR during the exercise session were analyzed to confirm that participants had achieved and maintained their HR within the targeted range (Supplementary Fig. 1).

### Fitbit

Participants were also outfitted with a Fitbit Charge 4 (Fitbit, San Francisco, USA) wristwatch, which collected data for the duration of the study. Fitbit’s proprietary algorithm was used to estimate participants’ resting HR. Reported resting HRs were taken from the first 7 days of study participation.

### Imaging

MRI and ^18^F-FE-PE2I PET scans were collected on separate days at baseline and after six months of exercise in the medication “off” state. Subjects were instructed to pause the exercise classes 1-2 weeks before the baseline and post-exercise PET scans to ensure that the measurements were not confounded by immediate exercise effects.

### PET

Dynamic PET scans were acquired on the High Resolution Research Tomograph scanner (Siemens/CTI, Knoxville, TN, USA) for 60 min starting with [^18^F]-FE-PE2I bolus injection over 1 min. After reconstruction and motion correction^52^, individual images were registered to MNI space by registering the PET image to the high-resolution MPRAGE image of the same subject and visit using SPM12^53^. The MPRAGE images were spatially normalized to MNI space^54^ and the estimated warping parameters were subsequently applied to the corresponding PET images. The registration and normalization quality of the MPRAGE and PET scans was checked visually. Three bilateral regions of interest were identified in each PET image; the putamen and caudate from the AAL template in the MNI space^55^, and a hand-drawn SN mask which was used in our previous studies^56–58^. For every region, a time-activity curve was extracted and modeled using the simplified reference tissue model (SRTM) with the cerebellum as reference region to estimate the regional binding potential (BP_ND_) and relative input parameter (R1). A voxel-level approach, still using the cerebellum curve as reference, was applied to normalized images to create parametric BP_ND_ maps^59^.

### MRI

Scans were collected in a 3.0 Tesla Siemens Prisma scanner using a 32-channel head coil. The following were collected: (1) high-resolution T1-weighted MPRAGE images (176 slices, voxel size: 1 mm^3^, FoV: 250 mm, matrix: 256 ×256, TR: 1900 ms, TE: 2.52 ms, TI: 900 ms, flip angle: 9°, scan time: 4 min 32 s), (2) 6-7 NM scans (magnetization transfer gradient echo sequences, FOV: 220 mm, 11 slices without gap aligned to the AC-PC line and the top slice 3 mm above the roof of the midbrain, voxel size: 0.4 ×0.4 ×2.5 mm, TR: 468 ms, TE: 3.7 ms, flip angle: 40°, scan time per scan: 2 min 53 s), and (3) high-resolution gradient echo sequences for QSM (FOV: 256 mm, aligned to the AC-PC line, voxel size: 0.5 ×0.5 ×1 mm, TR: 47 ms, TE1/∆TE: 6.10/4.02 ms, flip angle: 15°, scan time: 4 min 38 s).

The NM data were processed using an automated voxel-wise analysis pipeline that has been shown to be reliable and reproducible^60^. The FMRIB Software Library 6.0 (FSL)^61^ was used for motion correction and averaging, SPM12 was used for registration of the NM scans with the high-resolution MPRAGE anatomical scans^53^, and Advanced Normalization Tools (ANTS) for the spatial normalization of the MPRAGE scans to the standard MNI brain template^62^. The estimated warping parameters were then applied to the NM scans. The quality of the normalization of the NM scans to the MNI template for each subject was checked visually using SPM12. The SN pars compacta (SNc) mask in the MNI space defined by Pauli et al.^63^ was used as the region of interest. A hand-drawn in-house crus cerebri mask in the MNI space was used as the reference region. Voxel-level contrast ratio (CR) was calculated at voxel *j* according to Eq. (1):

1 $${{CR}}_{j}=\frac{{Intensity}\,{{SNc}}_{j}-{median}({{Intensity}}_{{crus}{cerebri}})}{{median}({{Intensity}}_{{crus}{cerebri}})}* 100$$

The CR for the entire SNc was calculated as the median value over all voxels.

For QSM scans, we followed the same motion correction, registration, and spatial normalization steps as for the NM scans. We used the same SNc template to extract the QSM values^63^. We used the automated QSM processing pipeline as described in Spincemaille et al.^64^. The total field was computed from the phase images by least-squares fitting, followed by background field removal using the projection onto dipole fields method to obtain the tissue field^65^. An R2* map was computed from the magnitude images using ARLO algorithm^66^, from which a ventricular cerebrospinal fluid (CSF) mask was created by thresholding. Finally, the morphology-enabled dipole inversion with automatic uniform CSF zero referencing (MEDI + 0) algorithm was used to reconstruct the susceptibility maps (in ppb) from the tissue field^67,68^. The QSM values were extracted from the SNc mask.

### Statistical analysis

The sample size was chosen based on reported annual decline in NM and reported positive effect sizes after exercise in PET^69–71^. Based on these reported estimates, a sample of 11 subjects would provide 80% power to detect a 5.6% increase in NM and a 10% increase in PET. Data analyses were conducted using JMP pro 16 (SAS Institute, Cary, USA). PET and NM-MRI data were analyzed using non-inferiority tests which compared the average of the observed individual change, defined as (post–pre)/pre * 100, to the reported percentage decreases in comparable PD cohorts. The observed change in DAT BP_ND_ over 6 months was compared to half the annual decline reported in Delva et al.^25^ (*n* = 27, age = 60.4 ± 9.7 years, H&Y = 2.0 ± 0.0, disease duration in years = 3.1 ± 1.0) and the observed change in NM CR over 6 months was compared to half the annual decline reported in Xing et al.^31^ (*n* = 46, age = 67.3 ± 8.7 years, H&Y = 2.0 ± 0.7, disease duration in years = 4.9 ± 1.8) (see supplementary material for details). Exploratory correlations between outcomes and demographics are in the supplementary material. Demographics are reported as median (range). Statistical outcomes are reported with their standard error and significance is defined as *p* < 0.05.

### Reporting summary

Further information on research design is available in the Nature Research Reporting Summary linked to this article.

### Supplementary information

Supplemental material

Reporting Summary

## Data Availability

The data that support the findings of this study are available from the corresponding author request.
