# Supplementary material for: Intense exercise increases dopamine transporter and neuromelanin concentrations in the substantia nigra in Parkinson’s disease
Source: NPJ Parkinsons Dis. 2024 Feb 9;10:34. doi: 10.1038/s41531-024-00641-1 (PMC10858031; doi:10.1038/s41531-024-00641-1)
Supplement: Supplementary file 1 — Supplemental material [file 41531_2024_641_MOESM1_ESM.pdf]

Supplemental Material

A representative example of the heart rate graphs used to evaluate if participants reached their target heart rates during the exercise sessions.

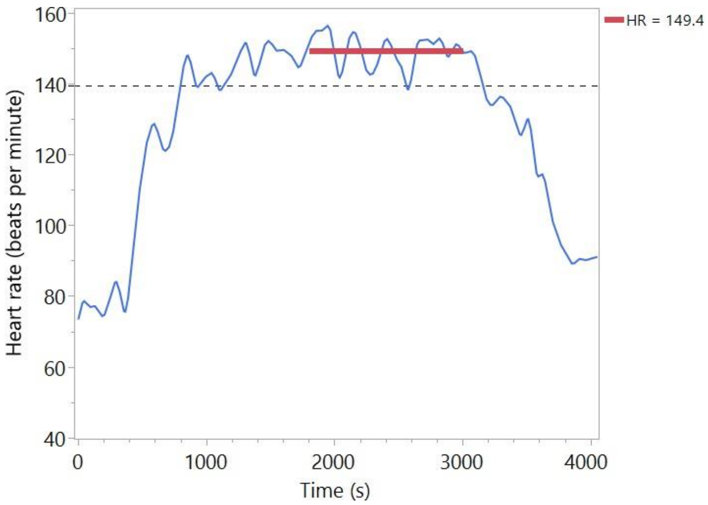

Supplementary Figure 1. Heart rate during an exercise session. The red bar shows the 20 minutes with highest average heart rate (here 149.4 beats per minutes), the dashed line represents 85% of the maximum heart rate of this subject.

Comparison with reference studies

Xing et al. divided the SN pars compacta (SNc) into ventral and dorsal tiers and reported the annualized rates of the NM contrast decay separately for both tiers. The annualized percent decay rates of the NM contrast for their PD cohort were -7.3 ± 19.1 (SNc ventral tier) and -5.3 ± 16.0 (SNc dorsal tier). Since we used the SNc as a whole (i.e., without dividing it into the ventral and dorsal tiers), we took the average change in NM contrast of those tiers: (7.3 + 5.3)/2 = 6.3. Of note, the annualized decay in the NM contrast was linear in the Xing et al. study. Therefore, we divided 6.3 by two (3.15) as an estimate of decay in 6 months.

We used the same approach for the DAT results. The annualized percent decay rates of DAT binding reported in the Delva et al. study for their PD cohort were the following: -7.8 ± 7.9 (Caudate), -10.7 ± 7.0 (Putamen), and -4.5 ± 13.2 (SN). We divided these mean values by two as an estimate of decay in 6 months.

Supplementary Table 1 summarizes the age, H&Y stage, and disease duration of the current and two comparator studies.

Supplementary Table 1. Averages with standard deviation. P-values of a Welch modified two-sample two-sided t-test comparing with the current study are reported in brackets.

	Current study (n=10)	Delva et al. 2022 (n=27)	Xing et al. 2022 (n=46)
Age (yr)	64.2 ± 5.2	60.4 ± 9.7 (0.13)	67.3 ± 8.7 (0.15)
H&Y stage	2 ± 0	2 ± 0 (1.00)	2 ± 0.7 (1.00)
Disease duration (yr)	2.0 ± 0.8	3.1 ± 1.0 (0.002)	4.9 ± 1.8 (<0.001)

Additional descriptive data of study cohort

Supplementary Tables 2, 3, and 4 show demographics, exercise data, and motor function test results for the entire intention-to-treat cohort (n=11).

Supplementary Table 2. Demographics for intention-to-treat cohort (n=11).

	Pre-exercise	Post-exercise
Age (years)	65.3 ± 6.0	
Sex	6 males, 5 females	
Disease duration (years)	1.9 ± 0.8	
Symptom onset side	6 right, 5 left	
Pre-exercise moderate-intensity exercise (hr/wk)	8.2 ± 4.6	
Pre-exercise moderate-intensity MET/kg/wk	31.2 ± 19.6	
H & Y	2.0 ± 0.0	2.0 ± 0.0
MDS-UPDRS I	8.5 ± 5.8	6.8 ± 4.1
MDS-UPDRS II	3.8 ± 2.4	3.3 ± 1.6
MDS-UPDRS III	25.8 ± 6.9	26.0 ± 8.6
MDS-UPDRS IV	1.0 ± 1.1	0.6 ± 0.9
MDS-UPDRS total	36.9 ± 12.8	36.7 ± 10.6
MoCA	28.3 ± 1.8	28.3 ± 1.1
STAI-T	36.8 ± 13.3	35.0 ± 11.9
BDI-II	7.7 ± 6.3	6.1 ± 4.9
Apathy	10.8 ± 6.5	8.6 ± 5.3
PDQ-39-SI	11.7 ± 6.6	9.4 ± 4.4
PFS-16	2.2 ± 1.2	2.0 ± 1.0
LEDD (mg)	250.0 ± 150.0	243.2 ± 189.1

Supplementary Table 3. Exercise data

# Completed exercise classes	88.8 ± 20.6
Heart rate (HR) data	
Resting HR (bpm)	64.7 ± 7.3
Max HR (bpm)	126.8 ± 13.1
% classes at > 80% max HR target	62.4 ± 36.1
% classes at > 70% max HR target	86.7 ± 22.7
Weekly surveys	
Motivation to exercise	3.9 ± 0.1
Satisfaction with class/trainer	3.9 ± 0.1
Satisfaction with own performance	3.5 ± 0.4
Intensity of class	3.6 ± 0.3

Bpm: beat per minute. Scale (0-4) for surveys: 0: very unmotivated, very unsatisfied, not intense at all and 4: very motivated, very satisfied, very intense

Supplementary Table 4. Motor function tests

	Pre-exercise	Post-exercise
5 times sit and stand (s)	9.7 ± 1.8	8.7 ± 1.6
360° turn (s)	2.6 ± 0.8	2.3 ± 0.4
Timed-up-and-go (s)	7.1 ± 1.6	6.7 ± 0.9
Climb up 1 flight of stairs (s)	4.9 ± 0.9	4.9 ± 0.7
2-min walk (m)	179.0 ± 37.7	185.0 ± 33.0

Supplementary Table 5 shows comparison of clinical survey scores of our cohort (n = 10) and the comparison with the normative mean.

Table 5. Clinical survey data

	Normative mean / Threshold	Pre-exercise	Post-exercise	P-value pre-exercise vs norm.*
MoCA (mild cognitive impairment)	<26	28.3 ± 1.8	28.3 ± 1.1	0.001
STAI-T (F/M)	>31.79 / >33.86	36.8 ± 13.3	35.0 ± 11.9	0.80
BDI-II (minimal depression)	>13	7.7 ± 6.3	6.1 ± 4.9	0.01
Apathy	>14	10.8 ± 6.5	8.6 ± 5.3	0.08
PDQ-39-SI	>31.6	11.7 ± 6.6	9.4 ± 4.4	<0.001
PFS-16	>2.95	2.2 ± 1.2	2.0 ± 1.0	0.04

* p-values were calculated with one-sample t-tests assessing the probability of observing our cohort's results if the true mean was at the normative mean / threshold.

Exploratory correlations

Exploratory analyses compared post- versus pre-exercise differences in DAT BP_{ND} and NM CR with post – pre differences in R1, MDS-UPDRS-III, and mood.

Fourteen exploratory correlations were assessed between the observed difference in FE-PE2I binding of all 3 regions and the SN CR by differences in MDS-UPDRS-III score, PDQ39-SI, R1, and QSM before and after exercise. No correlation was found to be significant (Supplementary Table 6), except for the correlation between the difference in FE-PE2I binding in the caudate and the difference in MDS-UPDRS-III scores before and after exercise (p = 0.0067, effect: -0.79, 95% CI: -0.95 to -0.32) (Figure 3).

Variable	by Variable	Correlation	Lower 95% CI	Upper 95% CI	# Subjects	P-Value
Diff BP caudate	Diff R1 caudate	-0.18	-0.73	0.50	10	0.61
Diff BP caudate	Diff MDS-UPDRS-III	-0.79	-0.95	-0.32	10	**0.007**
Diff BP caudate	Diff PDQ39-SI	-0.38	-0.82	0.33	10	0.28
Diff BP putamen	Diff R1 Putamen	0.27	-0.43	0.77	10	0.45
Diff BP putamen	Diff MDS-UPDRS-III	-0.42	-0.83	0.29	10	0.23
Diff BP putamen	Diff PDQ39-SI	-0.36	-0.81	0.35	10	0.31
Diff BP SN	Diff R1 SN	-0.12	-0.70	0.55	10	0.74
Diff BP SN	Diff MDS-UPDRS-III	-0.06	-0.67	0.59	10	0.86
Diff BP SN	Diff QSM SN	-0.24	-0.76	0.46	10	0.50
Diff BP SN	Diff PDQ39-SI	-0.53	-0.87	0.15	10	0.11
Diff CNR SNc	Diff R1 SN	0.13	-0.54	0.70	10	0.72
Diff CNR SNc	Diff MDS-UPDRS-III	0.21	-0.45	0.72	11	0.54
Diff CNR SNc	Diff QSM SN	-0.34	-0.80	0.37	10	0.34
Diff CNR SNc	Diff PDQ39-SI	-0.02	-0.61	0.59	11	0.96

Supplementary Table 6. Correlations and their 95% CIs per pairwise comparison. Diff = difference, defined as post- exercise – baseline values, SN = substantia nigra, SNc = substantia nigra pars compacta, BP = binding potential, CR = contrast ratio, # Subjects = number of subjects included for this correlation.

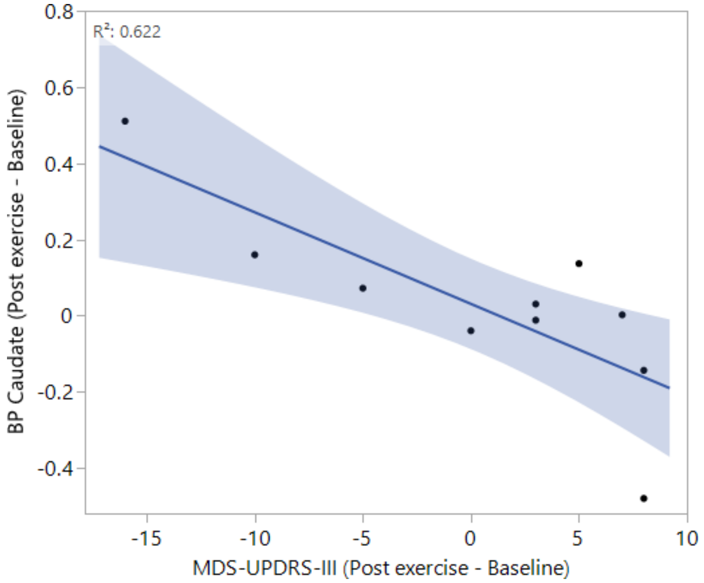

Supplemenatry Figure 2. Difference, defined as post- versus pre-exercise, in FE-PE2I BP_{ND} versus the difference in blinded MDS-UPDRS-III scores.
