# Supplementary material for: Intense exercise increases dopamine transporter and neuromelanin concentrations in the substantia nigra in Parkinson’s disease
Source: NPJ Parkinsons Dis. 2024 Feb 9;10:34. doi: 10.1038/s41531-024-00641-1 (PMC10858031; doi:10.1038/s41531-024-00641-1)
Supplement: Supplementary file 2 — Reporting Summary [file 41531_2024_641_MOESM2_ESM.pdf]

nature portfolio

Corresponding author(s): Bart de Laat

Last updated by author(s): Jan 15, 2024

Reporting Summary

Nature Portfolio wishes to improve the reproducibility of the work that we publish. This form provides structure for consistency and transparency in reporting. For further information on Nature Portfolio policies, see our Editorial Policies and the Editorial Policy Checklist.

Statistics

For all statistical analyses, confirm that the following items are present in the figure legend, table legend, main text, or Methods section.

n/a	Confirmed	
☐	☒	The exact sample size (*n*) for each experimental group/condition, given as a discrete number and unit of measurement
☐	☒	A statement on whether measurements were taken from distinct samples or whether the same sample was measured repeatedly
☐	☒	The statistical test(s) used AND whether they are one- or two-sided<br>*Only common tests should be described solely by name; describe more complex techniques in the Methods section.*
☒	☐	A description of all covariates tested
☐	☒	A description of any assumptions or corrections, such as tests of normality and adjustment for multiple comparisons
☐	☒	A full description of the statistical parameters including central tendency (e.g. means) or other basic estimates (e.g. regression coefficient) AND variation (e.g. standard deviation) or associated estimates of uncertainty (e.g. confidence intervals)
☐	☒	For null hypothesis testing, the test statistic (e.g. *F*, *t*, *r*) with confidence intervals, effect sizes, degrees of freedom and *P* value noted<br>*Give P values as exact values whenever suitable.*
☒	☐	For Bayesian analysis, information on the choice of priors and Markov chain Monte Carlo settings
☒	☐	For hierarchical and complex designs, identification of the appropriate level for tests and full reporting of outcomes
☐	☒	Estimates of effect sizes (e.g. Cohen's *d*, Pearson's *r*), indicating how they were calculated

Our web collection on statistics for biologists contains articles on many of the points above.

Software and code

Policy information about availability of computer code

Data collection	None.
Data analysis	None.

For manuscripts utilizing custom algorithms or software that are central to the research but not yet described in published literature, software must be made available to editors and reviewers. We strongly encourage code deposition in a community repository (e.g. GitHub). See the Nature Portfolio guidelines for submitting code & software for further information.

Data

Policy information about availability of data

All manuscripts must include a data availability statement. This statement should provide the following information, where applicable:

- Accession codes, unique identifiers, or web links for publicly available datasets
- A description of any restrictions on data availability
- For clinical datasets or third party data, please ensure that the statement adheres to our policy

The data that support the findings of this study are available from the corresponding author upon reasonable request.

Research involving human participants, their data, or biological material

Policy information about studies with human participants or human data. See also policy information about sex, gender (identity/presentation), and sexual orientation and race, ethnicity and racism.

Reporting on sex and gender	Recruitment was done irrespective of sex, gender was not taken into account. The small sample does not allow sex-specific analyses.
Reporting on race, ethnicity, or other socially relevant groupings	Recruitment was done irrespective of race, ethnicity, or other socially relevant groupings. None of these factors was used as a covariate in the analysis.
Population characteristics	Pre-exercise Post-exercise<br>Age (years) 64.2 ± 5.2<br>Sex 6 males, 4 females<br>Disease duration (years) 2.0 ± 0.8<br>Symptom onset side 5 right, 5 left<br>Pre-exercise moderate-intensity exercise (hr/wk) 8.4 ± 4.8<br>Pre-exercise moderate-intensity MET/kg/wk 32.3 ± 20.3<br>Weight (kg) 76.6 ± 14.5 74.6 ± 14.1<br>H & Y 2.0 ± 0.0 2.0 ± 0.0<br>MDS-UPDRS I 8.6 ± 6.1 6.5 ± 4.2<br>MDS-UPDRS II 3.5 ± 2.2 3.1 ± 1.6<br>MDS-UPDRS III 27.6 ± 4.4 27.0 ± 8.4<br>MDS-UPDRS IV 1.0 ± 1.2 0.6 ± 1.0<br>MDS-UPDRS total 37.6 ± 13.6 37.2 ± 11.0<br>MoCA 28.1 ± 1.8 28.2 ± 1.1<br>STAI-T 34.5 ± 11.4 34.5 ± 12.4<br>BDI-II 7.8 ± 6.6 5.7 ± 5.0<br>Apathy 10.9 ± 6.8 8.2 ± 5.4<br>PDQ-39-SI 11.8 ± 7.0 9.2 ± 4.6<br>PFS-16 2.1 ± 1.2 2.0 ± 1.1<br>LEDD (mg) 245.0 ± 157.1 215.0 ± 173.3
Recruitment	Recruitment was done through the exercise program that was used as the intervention in this study.
Ethics oversight	Yale University HIC

Note that full information on the approval of the study protocol must also be provided in the manuscript.

Field-specific reporting

Please select the one below that is the best fit for your research. If you are not sure, read the appropriate sections before making your selection.

☒ Life sciences ☐ Behavioural & social sciences ☐ Ecological, evolutionary & environmental sciences

For a reference copy of the document with all sections, see nature.com/documents/nr-reporting-summary-flat.pdf

Life sciences study design

All studies must disclose on these points even when the disclosure is negative.

Sample size	The sample size was chosen based on reported annual decline in NM and reported positive effect sizes after exercise in PET 62-64. Based on these reported estimates, a sample of 11 subjects would provide 80% power to detect a 5.6% increase in NM and a 10% increase in PET.
Data exclusions	Data from 1 subject was excluded due to lack of adherence to study protocol.
Replication	Detailed description of both the intervention, methods and analysis.
Randomization	Not applicable, all subjects were followed before and after the same intervention.
Blinding	Blinding was performed for the subjective clinical assessments.

Reporting for specific materials, systems and methods

We require information from authors about some types of materials, experimental systems and methods used in many studies. Here, indicate whether each material, system or method listed is relevant to your study. If you are not sure if a list item applies to your research, read the appropriate section before selecting a response.

Materials & experimental systems

n/a	Involved in the study
☒	☐ Antibodies
☒	☐ Eukaryotic cell lines
☒	☐ Palaeontology and archaeology
☒	☐ Animals and other organisms
☒	☐ Clinical data
☒	☐ Dual use research of concern
☒	☐ Plants

Methods

n/a	Involved in the study
☒	☐ ChIP-seq
☒	☐ Flow cytometry
☐	☒ MRI-based neuroimaging

Plants

Seed stocks

Report on the source of all seed stocks or other plant material used. If applicable, state the seed stock centre and catalogue number. If plant specimens were collected from the field, describe the collection location, date and sampling procedures.

Novel plant genotypes

Describe the methods by which all novel plant genotypes were produced. This includes those generated by transgenic approaches, gene editing, chemical/radiation-based mutagenesis and hybridization. For transgenic lines, describe the transformation method, the number of independent lines analyzed and the generation upon which experiments were performed. For gene-edited lines, describe the editor used, the endogenous sequence targeted for editing, the targeting guide RNA sequence (if applicable) and how the editor was applied.

Authentication

Describe any authentication procedures for each seed stock used or novel genotype generated. Describe any experiments used to assess the effect of a mutation and, where applicable, how potential secondary effects (e.g. second site T-DNA insertions, mosiacism, off-target gene editing) were examined.

Magnetic resonance imaging

Experimental design

Design type

This only refers to BOLD based MRI, which was not used in our study.

Design specifications

NA

Behavioral performance measures

NA

Acquisition

Imaging type(s)

Functional, NM, QSM

Field strength

3

Sequence & imaging parameters

(1) high-resolution T1-weighted MPRAGE images (176 slices, voxel size: 1 mm3, FoV: 250 mm, matrix: 256 x 256, TR: 1900 ms, TE: 2.52 ms, TI: 900 ms, flip angle: 9°, scan time: 4 min 32 s), (2) 6-7 NM scans (magnetization transfer gradient echo sequences, FOV: 220 mm, 11 slices without gap aligned to the AC-PC line and the top slice 3 mm above the roof of the midbrain, voxel size: 0.4 x 0.4 x 2.5 mm, TR: 468 ms, TE: 3.7 ms, flip angle: 40°, scan time per scan: 2 min 53 s), and (3) high-resolution gradient echo sequences for QSM (FOV: 256 mm, aligned to the AC-PC line, voxel size: 0.5 x 0.5 x 1 mm, TR: 47 ms, TE1/ΔTE: 6.10/4.02 ms, flip angle: 15°, scan time: 4 min 38 s).

Area of acquisition

See above.

Diffusion MRI ☐ Used ☒ Not used

Preprocessing

Preprocessing software

The NM data were processed using an automated voxel-wise analysis pipeline that has been shown to be reliable and reproducible 53. The FMRIB Software Library 6.0 (FSL) 54 was used for motion correction and averaging, SPM12 was used for registration of the NM scans with the high-resolution MPRAGE anatomical scans 46, and Advanced Normalization Tools (ANTS) for the spatial normalization of the MPRAGE scans to the standard MNI brain template 55. The estimated warping parameters were then applied to the NM scans. The quality of the normalization of the NM scans to the MNI template for each subject was checked visually using SPM12. The SN pars compacta (SNc) mask in the MNI space defined by Pauli et al. 56 was used as the region of interest. A hand-drawn in-house crus cerebri mask in the MNI space was used as the reference region.

Normalization

See above.

Normalization template

See above.

Noise and artifact removal | See above.

Volume censoring | See above.

Statistical modeling & inference

Model type and settings | Equivalence test.

Effect(s) tested | NA

Specify type of analysis: ☐ Whole brain ☒ ROI-based ☐ Both

Anatomical location(s) | See above.

Statistic type for inference (See Eklund et al. 2016) | NA

Correction | NA

Models & analysis

n/a	Involved in the study
☒	☐ Functional and/or effective connectivity
☒	☐ Graph analysis
☒	☐ Multivariate modeling or predictive analysis
